# Expanding the multicolor capabilities of basic confocal microscopes by employing red and near-infrared quantum dot conjugates

**DOI:** 10.1186/1472-6750-9-49

**Published:** 2009-05-22

**Authors:** Lara M Kingeter, Brian C Schaefer

**Affiliations:** 1 Department of Microbiology and Immunology, Uniformed Services University, 4301 Jones Bridge Road, Bethesda, MD 20814 USA; 2 Department of Pathology, Human Immune Therapy Center, University of Virginia Health System, Charlottesville, VA 22908 USA

## Abstract

**Background:**

Confocal microscopy is a widely employed methodology in cellular biology, commonly used for investigating biological organization at the cellular and sub-cellular level. Most basic confocal microscopes are equipped to cleanly discriminate no more than four fluorophores in a given sample, limiting the utility of this method for co-localization, co-expression, and other multi-parameter analyses. In this study, we evaluated the use of red and near-infrared emitting quantum dot staining reagents to expand the multi-parameter capabilities of basic confocal microscopes.

**Results:**

We modified a three-laser Zeiss Pascal confocal microscope by the addition of two band-pass filters and one long-pass filter for the detection of three different red to near-infrared quantum dot conjugates. We then performed direct comparisons between organic dye- and quantum dot-labeled detection reagents for the detection of subcellular structures. We found that the quality of staining was generally indistinguishable, although quantum dot reagents do have certain limitations, relative to organic dye conjugates. Using the modified Pascal system, three quantum dot conjugates, two organic dye conjugates, and one fluorescent protein, we demonstrated clean discrimination of six distinct fluorescent labels in a single sample.

**Conclusion:**

Our data demonstrate that nearly any basic confocal microscope can be modified by the simple addition of appropriate emission filters, allowing the detection of red and near-infrared quantum dot conjugates. Additionally, quantum dot- and organic dye-based secondary reagents can be successfully combined in complex intracellular staining experiments. Substantial expansion of the multi-parameter capabilities of basic confocal instruments can be achieved with a financial investment that is minimal in comparison to instrument replacement or upgrade with additional lasers.

## Background

Over the past 20 years, confocal microscopy has become a centrally important technique for the analysis of biological samples. By using a pinhole to exclude scattered light, confocal instruments can be used to optically section biological samples, producing 2- and 3-dimensional images with spatially resolved details at the sub-micron level. Beyond simply visualizing fluorescently labeled specimens, confocal microscopy has become a powerful tool for biologists in many disciplines for diverse applications, including establishing structure-function relationships at the cellular and tissue level, defining dynamic processes in living specimens, and for detection of close interactions between biological molecules at the subcellular level [1].

Most basic confocal microscopes are equipped with 2, 3, or 4 lasers, and are generally configured to detect one fluorophore per laser, giving a maximum detection of four distinct fluorescent labels in a single sample. There are several different factors that contribute to this limitation, including the fact that the most prevalent fluorescent probes are small organic molecules which have a small Stoke's shift. Thus, with few exceptions, each fluorescent dye in an experiment requires a distinct laser for excitation, and the emission spectrum is slightly red-shifted, relative to the excitation wavelength. As a result, the number of proteins or cell structures that can be imaged concurrently is quite restricted (reviewed in [1] and [2]).

Quantum dot (Qdot)-coupled detection reagents offer an opportunity to expand the capabilities of basic confocal instruments. Qdots are semi-conductor nanocrystals consisting of a CdSe core and a surface chemistry treatment which allows the Qdot to be coupled to proteins [3]. A striking advantage of Qdots over most organic fluorophores involves their long fluorescence half-life and high resistance to photobleaching, allowing them to be imaged extensively with minimal loss of signal [3]. Qdots have several additional properties which make them attractive for imaging applications, including a wide excitation spectrum, a narrow emission spectrum, and a long Stoke's shift. The physics governing Qdots fluorescence is such that the emission wavelength is determined by the size of the Qdot. Consequently, larger Qdots have longer emission wavelengths. Importantly, all Qdots share overlapping excitation spectra, with maximal excitation by ultraviolet (UV) wavelengths, meaning that the Stoke's shift for red and infrared Qdots spans hundreds of nanometers, which clearly distinguishes these fluorophores from organic dyes [4]. An additional consequence of this Qdots property is that all Qdots can be efficiently excited by a single laser in the UV to blue region of the spectrum [3]. Commercially produced Qdots reagents are now available with defined emission wavelengths that extend from green to the near-infrared emission wavelengths.

The physical properties of Qdots, predominantly their large size (diameters in the nanometer range [5]), dictate that numerous antibodies are coupled to a single Qdot. In contrast, when labeling with organic dyes (which are small, relative to an antibody), many dye molecules are coupled to an individual antibody. Thus, Qdot coupled antibodies are both much larger and have many more ligand binding sites than organic dye coupled antibodies. It is therefore reasonable to expect that these reagents may behave quite differently for applications such as the staining of detergent permeabilized cells.

In principle, Qdot-labeled staining reagents can be combined with organic dye conjugates and/or fluorescent proteins to expand the number of parameters detected in a single fluorescently labeled sample. Given that quantum dot reagents with red and near-infrared (IR) emissions are very bright and cover a region of the spectrum that is under-utilized on many basic confocal systems, staining reagents that are coupled to red and near-IR quantum dots represent a particularly attractive strategy to expand the multi-parameter capabilities of confocal microscopes. However, due to the large size of quantum dot conjugates and their multivalency, these reagents perform poorly for one-to-one ligand-to-target binding [3]. Thus, Qdot-labeled reagents can be expected to behave differently from organic dye-labeled conjugates in fluorescent labeling experiments. We thus initiated a study to compare the quality of images obtained from Qdot- and organic dye-labeled conjugates for the staining of fixed, detergent permeabilized cells, and to test the suitability of red and near-IR Qdot reagents for performing multi-parameter fluorescence labeling experiments in combination with organic dye-based detection reagents. Together, our data demonstrate that, with simple and inexpensive modifications to a basic confocal microscope, red and near-IR Qdot-labeled conjugates can readily be combined with conventional fluorophores, increasing the number of labeled structures that can be detected and cleanly discriminated in a single experiment.

## Results and Discussion

To test the use of Qdots in a basic confocal system, we employed a Zeiss LSM5 Pascal system equipped with a 25 mW 405 nm diode laser, a 25 mW argon laser tunable to 458 nm, 488 nm or 514 nm, a 5 mW 543 nm Helium/Neon laser, and two fluorescence detector channels, which utilize Hamamatsu R6357 photomultiplier tubes (PMTs). With this system, three-color detection of organic fluorophores in combinations such as DAPI, Alexa 488 and Alexa 555 (405 nm, 488 nm, and 543 nm excitation) or combinations of fluorescent proteins and organic probes, such as CFP, YFP, and Alexa 555 are easily achieved. However, acquisition of four or more fluorescence parameters from a single sample employing organic dyes and/or fluorescent proteins is quite difficult on this system, due to insufficient separation of excitation and/or emission spectra.

**Figure 1:**
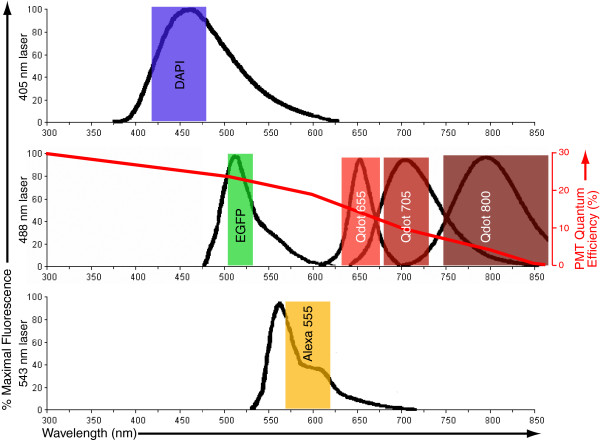
**Filter configuration and data acquisition strategy for 6-parameter imaging experiments**. Graphs show emission spectra of organic dye and Qdot reagents employed in this study. The spectra are grouped with the laser line used for excitation (405 nm, top; 488 nm, middle; 543 nm, bottom). Colored bands indicate the approximate range of emissions transmitted by the filters (see also Table 1). In the middle graph, the red line and red axis indicates the approximate quantum efficiency of the Hamamatsu 6357 PMT through the displayed wavelength range. Spectra are based on manufacturer's supplied data from Invitrogen (DAPI and Qdots) and Clontech (EGFP), and PMT quantum efficiency information is based on technical data from Hamamatsu.

To expand the multi-parameter capabilities of our LSM5 Pascal, we added additional emission filters to allow combinatorial detection of red- and near infrared-emitting Qdots (End users can easily add or change emission filters in the LSM5 Pascal scan head, following brief training by a Zeiss service engineer). Specifically, we added 655/40 nm and 705/50 nm band pass filters and a 745 nm long pass filter (all from Chroma Technologies, Brattleboro, VT) to enable detection of Qdots with fluorescence emissions at 655 nm, 705 nm and 800 nm, respectively (Fig. 1 and Table 1).

**Table 1:** Confocal imaging parameters.

**Fluorophore**	**Filter Set**	**Pinhole Diameter**	**Laser (nm)/Transmission (%)**	**Detector Gain**
DAPI	BP 420–480	74	405/4.1	766
GFP	BP 505–530	82	488/46.6	765
Alexa 555	BP 570–620	94	543/16.6	542
Qdot 655	BP 635–675	106	488/34.7	618
Qdot 705	BP 680–730	106	488/34.7	578
Qdot 800	LP 745	82	488/46.6	649

**Figure 2:**
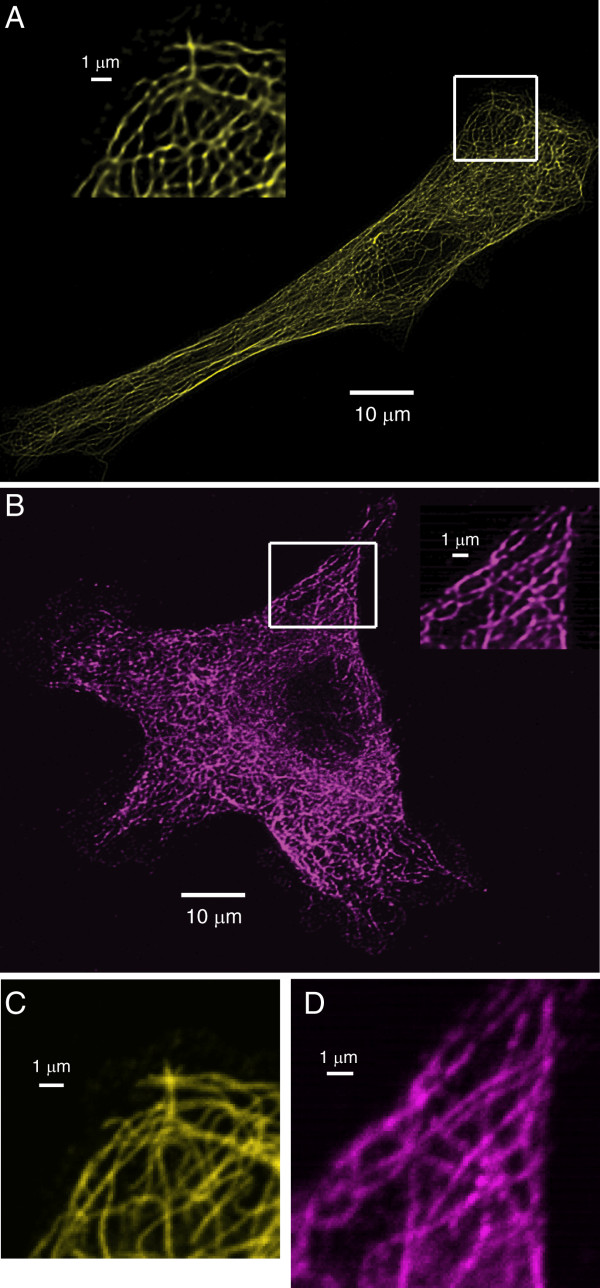
**Alexa 555 and Qdot 655 secondary antibodies produce images of similar quality**. Fibroblasts were stained with anti-α-tubulin antibody, followed by secondary antibodies coupled to Alexa 555 (A) or Qdot 655 (B). Insets show magnification of outlined region. Images in (A) and (B) are maximal projections of deconvolved z-stack data. Images in (C) and (D) show single xy planes of the inset regions from (A) and (B), prior to deconvolution.

Details of the imaging parameters used during the collection of data for Fig. 2. Amplifier offset and amplifier gain settings were constant throughout (0.1 and 1, respectively).

Evaluation of the Pascal's fluorescence detection hardware suggested that fluorescence emission from each of these Qdots could be detected. Indeed, the Pascal's Hamamatsu R6357 PMTs maintain high quantum efficiency (QE) (Fig. 1) through most of this spectral range (10–20% in the 600–700 nm range; maximal QE for this PMT is approximately 30%). Although the Pascal's PMTs have <5% QE near 800 nm, we predicted that the extremely bright fluorescence from Qdot 800 reagents could still be successfully detected, with adequate cell labeling. The total cost of these filter upgrades was less than $800.

To determine whether or not Qdot conjugated antibodies behave comparably to organic dye conjugated antibodies for the staining of intracellular structures in fixed, detergent permeabilized cells, NIH/3T3 fibroblasts were grown on glass coverslips, fixed with paraformaldehyde, permeabilized with a 0.2% Triton X-100 solution, and stained with a monoclonal antibody against α-tubulin. Duplicate coverslips were then stained with either an organic dye-labeled secondary antibody (Alexa 555 goat anti-mouse IgG_1_) or a Qdot-labeled secondary antibody (Qdot 655 goat anti-mouse IgG). Fluorescence data were acquired as z-stack images on our modified Zeiss LSM5 Pascal, and data were further processed by digital deconvolution and projection into a 2-dimensional image. As shown in Fig. 2, the Alexa 555-conjugated secondary antibody (Figs. 2A, C) and the Qdot-conjugated secondary antibody (Figs. 2B, D) generally yielded images of similar quality, with individual microtubules labeled sharply and contiguously throughout the majority of the cell, both when comparing deconvolved z-stack maximal intensity projection images (Fig. 2A–B), and when comparing single xy planes, prior to deconvolution (Figs 2C–D).

The one consistent difference in labeling that we noted was that the signal-to-noise ratio of the Qdot 655 labeled samples was lower than observed with the Alexa 555 labeled cells, which may account for the weak and variable staining of Qdot 655-labeled microtubules at sites of adhesion of the fibroblasts to the coverslip (compare the margins of adhesion sites in Fig. 2B and Fig. 2A). However, it is important to note that this result is clearly due, at least in part, to the reduced quantum efficiency of the Hamamatsu R6357 PMT in the red and near IR region of the spectrum and the narrower band pass filter used for the collection of Qdot 655 vs. Qdot 555 fluorescence emissions (40 nm vs. 50 nm, respectively) (Fig. 1 and Table 1). Overall, the data in Fig. 2 demonstrate that Qdot-coupled and organic dye-coupled antibodies behave comparably for the staining of intracellular antigens. However, bright organic dyes that fluoresce in the region of maximal PMT quantum efficiency may be more appropriate for the detection of low abundance antigens or for maximal resolution of fine structural details.

We next performed a multi-parameter labeling experiment to test the feasibility of separation of six distinct fluorescent signals on our modified LSM5 Pascal system. For this experiment, we used a D10 T cell line expressing PKCθ-GFP during interactions with a fibroblast cell line (3T3-APC) engineered to specifically activate D10 through its T cell receptor (see Methods for further details). We chose this particular combination of cell lines because we have previously characterized the antigen-stimulated redistribution of intracellular proteins (including PKCθ) in the D10 line [6], and because D10 T cells and fibroblasts have very different sizes and morphologies, allowing us to confirm the identity of each cell type independently of fluorescence data. The 3T3-APC cells were grown overnight on sterile coverslips, and then labeled with the Qtracker^® ^Q800 Cell Labeling Kit, which results in accumulation of the Qdot 800 label in cytosolic vesicles. Next, D10 T cells expressing a PKCθ-GFP fusion protein were added to the Qtracker-labeled 3T3-APC cells. The coverslips were incubated 10 min at 37°C to facilitate T cell interactions with 3T3-APC cells and consequent activation of the D10 T cells. The cells were then fixed, permeabilized, and stained with biotin-XX-phalloidin for the detection of F-actin, and with primary antibodies to α-tubulin and RelA, for the detection of microtubules and the NF-κB transcription factor, respectively. We then added fluorescent secondary reagents: Qdot 705 Streptavidin for the detection of biotinylated phalloidin, Qdot 655 anti-rabbit IgG for the detection of the polyclonal anti-RelA antibody, and Alexa 555 Anti-IgG_1 _for the detection of the monoclonal anti-α-tubulin antibody. Immediately prior to mounting, nuclear DNA was labeled with DAPI. Slides were visualized using our modified Zeiss LSM5 Pascal confocal microscope, employing the imaging parameters outlined in Fig. 1 and detailed in Table 1. Fluorescent z-stack data were deconvolved and flattened as described in Methods.

**Figure 3:**
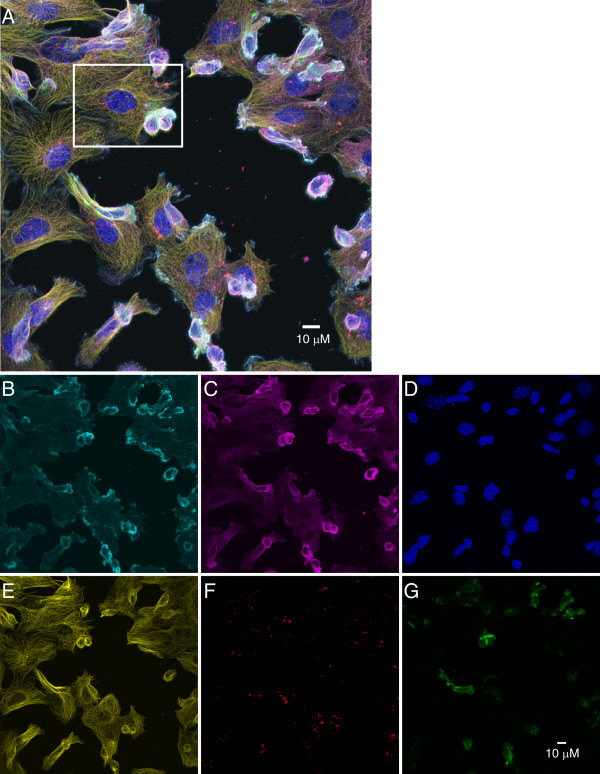
**Qdot reagents expand the number of fluorophores detectable in a single experiment**. 3T3-APC/T cell conjugates were stained with a combination of Qdot reagents and organic fluorophore reagents. Panel (A) shows the overlay of all 6 fluorophore signals; the indicated region is magnified and displayed in Figure 4. The remaining panels show the individual fluorophore signals, (B) Qdot 705-phalloidin, (C) Qdot 655-RelA, (D) DAPI, (E) Alexa 555-tubulin, (F) Qdot 800-Qtracker, and (G) PKCθ-GFP.

**Figure 4:**
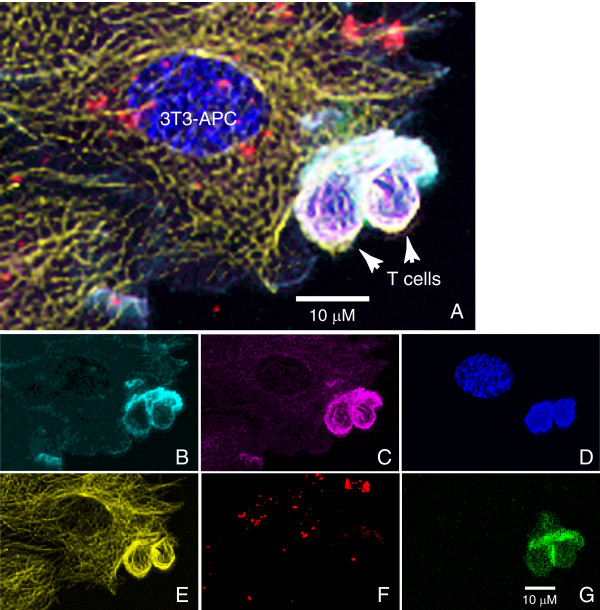
**Details of 3T3-APC/T cell interaction showing distribution of cellular proteins and structures**. Inset region from Figure 3 is magnified. Overlay image is in (A) and panels (B-G) show the same individual fluorophores as in Fig. 3.

As shown in Figs 3 and 4, we were able to clearly separate the signals from the six fluorescent probes used in this experiment. For each stain, the fluorescent signals were observed in the expected cell type(s) and subcellular distributions, with minimal cross-talk between channels. Specifically, F-actin was found in a membrane-proximal region of the cytosol, with particular concentration in the T cells at the region of junction with the stimulatory 3T3-APC cells [7] (Figs. 3B, 4B). RelA was found mostly in the cytosol of both the T cells (substantial nuclear translocation does not occur by 10 min post-stimulation [8]) and the 3T3-APC fibroblasts (Figs. 3C, 4C). DAPI (Figs. 3D, 4D) and anti-α-tubulin (Figs. 3E, 4E) labeled the nuclei and microtubules of both cell types, respectively. The Qtracker 800 label was found exclusively in the fibroblasts (Figs. 3F, 4F), and the PKCθ-GFP signal was detected only in the D10 T cells (Figs. 3G, 4G), with the expected enrichment at the site of contact with the 3T3-APC cells [6]. Together, these data demonstrate that with the addition of three additional filters to the LSM5 Pascal confocal and through the use of red and near-infrared Qdot labeling reagents, six distinct fluorescent labels can be readily detected and cleanly discriminated from each other.

Notably, due to the rather broad peak of the emission spectrum for Qdot 705, particularly in the near-infrared region of the spectrum, there is some signal bleed-through of the Qdot 705 signal into the Qdot 800 channel, with the specific filter design that we employed (see Fig. 1). For our experiment, use of the Qtracker 800 reagent produced a very intense Qdot 800 signal, such that the Qdot 705 signal bleed through was easily eliminated by raising the lower signal threshold in the Qdot 800 channel. As with any fluorescent staining protocol, it is imperative to empirically determine the degree of signal cross-talk and the intensity of staining in each channel, via analysis of singly-labeled samples imaged in all fluorescent channels, prior to attempting a combined labeling/imaging experiment.

Although we found that the Qdot-conjugated secondary antibodies yielded data of similar quality to organic dye labeled secondary reagents, we did note certain limitations of the Qdot labels in the course of our study. Firstly, we found that good labeling with the Qdot-conjugated antibodies was strictly dependent on use of the manufacturer's suggested blocking and staining buffers. For example, Qdot labeling was quite poor when serum was used as the blocking reagent in the secondary antibody labeling step, rather than albumin (data not shown). In contrast, excellent results were obtained with the organic dye labeled secondary reagents under either condition. Thus, for multi-parameter fluorescence microscopy experiments in which Qdots- and organic dye-based staining reagents are combined, it is very important to use the blocking and staining buffers that are optimal for the Qdots reagents.

Additionally, the cost of Qdot secondary reagents is considerably higher than organic dye labeled secondaries, when one considers the cost per staining reaction. Comparing Alexa 555 anti-mouse to Qdot 655 anti-mouse (both from Invitrogen, Carlsbad, CA; catalog numbers A21424 and Q11022MP), the current list prices are $172 for 500 μL and $191 for 100 μL, respectively. Given that the respective working concentrations are 1:500 and 1:100 and assuming a staining reaction volume of 200 μL, the cost per stain is $172/(500/0.4) = $0.14 for Alexa 555 anti-mouse vs. $191/(100/2) = $3.82 for Qdot 655 anti-mouse. Thus, Qdot-coupled secondary antibodies are approximately 27-fold more costly than comparable organic dye-labeled antibodies. Furthermore, we have observed that the useful shelf lives of the Qdot secondary reagents are generally limited to their stated expiration dates (0.5 – 1 yr beyond the date of purchase), presumably reflecting a gradual uncoupling between the Qdots and their antibody conjugates. This limitation represents a further increase in the effective per stain cost of quantum dot reagents for those laboratories that use such reagents only occasionally. In contrast, we have found that organic dye coupled secondaries are generally extremely stable for many years, with proper storage. Thus, Qdot reagents have specific disadvantages relative to organic dye-based reagents for routine intracellular staining applications. However, when considered in the context of the substantial potential benefits of the red and near IR quantum dots for multi-parameter confocal imaging, we view the above limitations as minor and manageable.

## Conclusion

Through the simple addition of appropriate emission filters, basic confocal microscopes can be modified for detection and discrimination of multiple red and near-infrared Qdot reagents. Incorporating Qdot-conjugated antibodies into staining protocols is a technically simple procedure that allows the utilization of existing lasers and PMT tubes (or CCD cameras), while dramatically expanding the multi-parameter imaging capabilities of this instrumentation at minimal expense. In summary, we have shown that a generalizable and easily implemented upgrade can greatly augment the capabilities of pre-existing confocal instrumentation in many laboratories and core facilities. We have also established staining conditions that yield optimal signal-to-noise and overall staining quality when Qdot- and organic dye-based reagents are used in multi-parameter labeling experiments.

## Methods

### Cell lines and staining reagents

NIH/3T3 cells were purchased from the ATCC. The 3T3-APC cell line was prepared by infecting MEF-3T3 Tet-Off cells (Clontech) with a tetracycline-inducible retroviral vector encoding IA^k^β with a covalently linked peptide from chicken conalbumin (HRGAIEWEGIES; the stimulatory ligand for the D10 T cell clone) and the IA^k^α chain. The resulting polyclonal population was subcloned by limiting dilution to produce a cell line with IA^k^-conalbumin peptide expression that was well regulated by tetracycline withdrawal (data not shown). The subcloned line was then further infected with pE retroviral vectors encoding murine ICAM-1, CD48 and ICOS-L [9], followed by selection with G418, hygromycin and zeocin, respectively. 3T3-APC cells were maintained in DMEM (Invitrogen) supplemented with 10% fetal bovine serum. The D10-PKCθ-GFP cell line (a CD4^+ ^T cell clone stably expressing PKCθ-GFP) was maintained in EHAA media (Invitrogen) as previously described [6].

Antibodies and staining reagents were as follows: mouse anti-α-tubulin (DM1A, 2 μg/mL, Sigma-Aldrich, St. Louis, MO); rabbit anti-RelA (SC372; 1 μg/mL, Santa Cruz Biotechnology, Santa Cruz, CA). Additionally, we used the following reagents from Invitrogen (Carlsbad, CA): biotin-XX-phalloidin (2.5 U); Alexa 555 goat anti-mouse IgG_1 _(4 μg/mL); Qdot 655 goat anti-mouse IgG (0.02 μM); Qdot 655 goat anti-rabbit IgG (0.01 μM) and Qdot 705 Streptavidin (0.01 μM); Qtracker^® ^800 Cell Labeling Kit; DAPI (0.094 μM).

### Q800 Qtracker labeling of 3T3-APCs

Glass coverslips (18 mm square, #1 thickness) were dipped in ethanol and passed briefly over a Bunsen burner flame to sterilize. Coverslips were then transferred into the wells of 6-well tissue culture plates, and 2 mL of 3T3-APCs (1 × 10^5 ^total cells) in DMEM were added to each well. Cells were cultured overnight at 37°C in a humidified 5% CO_2 _incubator. For each coverslip, Q800 Qtracker^® ^and Carrier Reagent were mixed 1:1 (0.5 μL of each, per coverslip) in an Eppendorf tube, followed by 5 min incubation at ambient temperature. Next, 200 μL of complete DMEM was added to the Qtracker mix, pipetting several times to mix. Medium from a well containing 3T3-APCs was aspirated, and the Qtracker mix was added to the coverslip, forming a cushion of liquid resting on the coverslip. Cells were incubated for 45 min at 37°C in a humidified 5% CO_2 _incubator, followed by two washes in 1× PBS + 0.1% NaN_3 _(PBS-azide).

### Preparation of T cell/3T3-APC conjugates

IA^k^-conalbumin peptide expression was induced in 3T3-APC cells by withdrawal of doxycycline at least 16 hr prior to addition of T cells. Following Q800 Qtracker labeling, 1 × 10^5 ^D10-PKCθ-GFP T cells in 200 μl of EHAA media were added to 3T3-APC cells. T cell-fibroblast conjugation and T cell activation was induced by 10 min incubation at 37°C in a 5% CO_2 _incubator.

### Antibody staining

NIH/3T3 fibroblasts or T cell/3T3-APC conjugates were fixed by 10 min incubation at room temperature (RT) with 2 mL/well of paraformaldehyde solution (3% paraformaldehyde, 3% sucrose, 1× PBS, pH 7.6). Following two washes with PBS-azide, cells were treated for 10 min at RT with 2 mL/well of permeabilization buffer (0.2% Triton X-100 in PBS-azide). After two washes with PBS-azide, samples were treated for 45 min at RT with Qdots block solution (6% bovine serum albumin, 10% goat serum, 0.02% sodium azide, in 1× PBS), applied as a cushion of liquid (200 μL) on top of each coverslip.

To compare Qdots secondary reagents with organic dye-labeled secondaries, fixed and permeabilized NIH/3T3 cells were stained with mouse α-tubulin for 4 hr at RT, followed by Qdot 655 goat anti-mouse IgG or Alexa 555 goat anti-mouse IgG_1 _for 1 hr at RT (Fig. 2). All primary staining reagents were diluted in Qdots block solution, and secondary reagents were diluted in Qdots 2° buffer (6% BSA in 1× PBS). For labeling of T cell/3T3-APC conjugates, biotin-XX-phalloidin and antibodies to α-tubulin and RelA were added in a volume of 200 μL per coverslip and incubated for 4 hour at RT. Coverslips were washed twice with PBS:azide. Next, Alexa 555 anti-IgG_1_, Qdot 655 goat anti-rabbit, and Qdot 705 Streptavidin were added in a volume of 200 μL per coverslip and incubated for 1 hr at RT. Following two washes with 0.2% Triton X-100 in PBS-azide, DAPI diluted in Qdots 2°buffer was added to each coverslip in a volume of 200 μL and incubated for 10 min at RT. Coverslips were washed once in PBS-azide and mounted on glass microscope slides. The mount solution (10 μL/coverslip) consisted of 1 mg/mL p-phenylenediamine in 90% glycerol, pH 8.5. Excess mount solution was absorbed with filter paper and coverslip edges were sealed using clear nail polish. Slides were stored at -20°C in the dark prior to confocal imaging.

### Confocal microscopy and data processing

Fluorescent samples were visualized on a Zeiss LSM5 Pascal confocal system (built around a Zeiss Axiovert 200 M inverted microscope), using a 40× 1.3 N.A. oil objective and employing the imaging parameters listed in Table 1. Emission filter modifications to the Pascal are described in Results and Discussion. Data were collected as z-stacks with 20 planes and 0.47 μM spacing between each plane. Image deconvolution was performed with AutoDeblur Gold CWF (X1.4.1, Media Cybernetics), using a constrained iterative algorithm. The deconvolved z-stack data was then imported into MetaMorph (v. 6.1r0, Universal Imaging Corp.) and flattened using a maximal projection algorithm. For the data shown in Fig. 3, Adobe Photoshop (v. 7.0.1, Adobe Systems Inc.) was used to overlay data obtained from each fluorescent channel. Specifically, the fluorescence data from each channel was pasted into a distinct layer in a single Photoshop image, and the empty "Background" layer was deleted. The "screen" blending mode was selected for each channel to combine the fluorescent colors in each pixel. The final overlay image represents the blended fluorescence data for all six channels.

## Authors' contributions

LMK performed all staining and confocal microscopy experiments and protocol optimizations. BCS designed the study and made the 3T3-APC cell line. LMK and BCS wrote the manuscript.
